# Gender-Related Issues in the Management of Low-Back Pain: A Current Concepts Review

**DOI:** 10.3390/clinpract13060122

**Published:** 2023-10-30

**Authors:** Davide Bizzoca, Giuseppe Solarino, Alessandro Pulcrano, Giovanni Brunetti, Anna Maria Moretti, Lorenzo Moretti, Andrea Piazzolla, Biagio Moretti

**Affiliations:** 1 UOSD Vertebral Surgery, AOU Consorziale Policlinico di Bari, 70124 Bari, Italy; dott.piazzolla@gmail.com; 2 Ph.D. Course in Public Health, Clinical Medicine and Oncology, Department DiMePre-J, University of Bari “Aldo Moro”, 70124 Bari, Italy; 3 Orthopedics Unit, Department of Translational Biomedicine and Neuroscience “DiBraiN”, School of Medicine and Surgery, University of Bari, General Hospital, 70124 Bari, Italy; giuseppe.solarino@uniba.it (G.S.); biagio.moretti@uniba.it (B.M.); 4 Department of Pneumology, Santa Maria Hospital, Via De Ferrariis 18/D, 70124 Bari, Italy

**Keywords:** low-back pain, spine degenerative diseases, spinal stenosis, degenerative disc disease, lumbar intervertebral disc herniation, spondylolisthesis, vertebral instability

## Abstract

Background: Low back pain (LBP) is an emerging disease. This review aims to investigate the role of gender-related factors in the diagnosis, clinical, and surgical management of LBP. Methods: From January 2002 to March 2023, EMBASE, SCOPUS, OVID-MEDLINE, Google Scholar, PubMed, and Web of Science were searched to identify relevant papers for further analysis. Results: Fifteen papers were included in this review. Sex- and gender-related differences were analyzed regarding the following points: (1) LBP epidemiology; (2) LBP physiopathology; (3) conservative management of LBP; (4) major vertebral surgery for LBP. The conservative treatment of LBP highlights that women claim services later in terms of poorer health status than men. In the postoperative phase, female patients show worse LBP, quality of life, and disability, but equal or greater interval change, compared with male patients complaining of lumbar degenerative disease. Conclusions: LBP epidemiology and clinical outcomes, following conservative and surgical management of patients complaining of back pain, might depend on both sex- and gender-related factors. It is mandatory to assess gender-related indicators in patients referred to LBP and address them to improve their clinical outcomes and quality of life.

## 1. Introduction

Low back pain (LBP) is an emerging cause of pain and disability, whose prevalence has been rising in the last twenty years due to population expansion, the general population aging, and the worldwide pandemic of obesity [1,2,3,4,5,6]. LBP, defined as pain arising on the posterior aspect of the body in the area between the lower margin of the 12th rib and the gluteal region, in the general population has a point prevalence of 12–33% [2,3,4,5,6]. Almost 50% of LBP cases resolve within one to two weeks, and 90% will resolve within six to 12 weeks.

It occurs in all age groups, from childhood to old age, in both low- and high-income countries.

LBP could be differentiated into two main types: non-specific and specific. Non-specific LBP accounts for 90% of the cases, whereas only 5 to 10% of the cases are due to a specific cause [7]. The most common causes of LBP include vertebral fractures and their sequelae, axial spondylarthritis, tumors, infections, and intra-abdominal causes [7].

Sex- and gender-related factors have a relevant impact on the health status throughout the lifespan and influence disease pathogenesis, the reaction to pharmacologic and surgical treatment, and clinical outcomes [3].

Sex indicates the biological differences between males and females, mainly including reproductive/sexual anatomy, hormone level, gene expression, and cyclic variation, that involve different physiologic and anatomic features [3,4].

Gender is a more complex and multidimensional concept involving different non-biological variables, including education level, sociocultural differences, psychological aspects, economic status, drug assumption, lifestyle, comorbidities, and religious beliefs [3,4].

Recent studies suggest that gender plays a significant role in low back pain’s prevalence, intensity, and management [3,4].

Several studies have found that low back pain affects women more frequently than men [3]. According to the Global Burden of Disease Study in 2017, low back pain was the leading cause of disability among women worldwide [3,4]. This higher prevalence in women may be attributed to factors such as hormonal changes during menstruation, pregnancy-related changes, and anatomical differences [4].

Hormonal fluctuations, such as those occurring during the menstrual cycle, can contribute to increased pain sensitivity in women. Research suggests that changes in hormone levels, particularly estrogen and progesterone, affect pain perception and response [3,4]. Moreover, hormonal changes during pregnancy can lead to biomechanical alterations in the spine and pelvis, potentially causing low back pain [3].

Sociocultural factors also play a significant role in gender disparities relating to low back pain [3]. In many societies, traditional gender roles and expectations often dictate women’s responsibilities, including household chores, child-rearing, and caregiving [4]. These activities can increase the risk of musculoskeletal strain and injuries, leading to low back pain [3,4]. Moreover, societal pressures and cultural norms may discourage women from seeking appropriate medical attention and self-care, resulting in delayed treatment and progression of the condition [3,4,5,6].

Certain occupations and work-related activities may also contribute to gender disparities in low back pain. Jobs that require heavy lifting, prolonged sitting, or repetitive movements can increase the risk of developing musculoskeletal disorders, including low back pain [6].

Gender-related differences may also be observed in the healthcare setting [3,4,5,6]. Several studies have highlighted disparities in the diagnosis, treatment, and management of low back pain between men and women [3,4,5,6]. These disparities may be attributed to clinician bias, women’s reporting of pain, and societal expectations of gendered pain experiences [5].

This review aims to depict the importance of gender-related differences in the diagnosis, clinical, and surgical management of LBP.

## 2. Materials and Methods

### 2.1. Review Procedure

The review of the literature was conducted in 3 steps. The first step was a scoping literature search performed on papers published from January 2002 to March 2023 by two authors, tutored by another reviewer, using the following databases: SCOPUS, MEDLINE, EMBASE, Web of Science, OVID, PubMed, and Google Scholar. An initial pool of potentially relevant studies focused on sex- and gender-related issues in the diagnosis and management of LBP was selected.

The following terms were used in the search strategy: ((“gender-related differences” [MeSH Terms] OR “sex-related differences” [All Fields]) OR (“gender indicators” [MeSH Terms] OR “sex” [All Fields])) AND (“low back pain” [MeSH Terms] OR (vertebral pain [All Fields])).

### 2.2. Inclusion/Exclusion Criteria

Inclusion criteria were prospective and retrospective clinical trials focused on sex- and gender-related factors in patients complaining of LBP and papers written in English. Exclusion criteria: case reports, clinical reviews, and systematic reviews.

### 2.3. Data Extraction

The second step consisted of revising the included records to select studies dealing with sex- and gender-related factors in the diagnosis and clinical or surgical management of spine diseases causing LBP. The database search provided a total of 48 studies that could be potentially included in the review. After removing duplicates, 32 studies remained. Of these, 19 studies were discarded after reading titles and reviewing abstracts. Two additional abstracts were identified by checking the references of the relevant papers. A total of 15 articles were finally included in this present review.

## 3. Results

### 3.1. LBP Epidemiology

LBP can be differentiated, from a chronological point of view, into three main subtypes (acute, sub-acute, and chronic) based on symptoms duration and onset [6,7]. Acute LBP lasts less than 4 weeks, subacute back pain lasts 4 to 12 weeks, and chronic back pain lasts more than 12 weeks [6,7].

The prevalence of LBP is lower in males compared to females [8]. Female patients also suffer from different chronic and painful musculoskeletal system conditions in greater numbers than men because of their anatomy and hormonal levels [9].

Sex differences in pain perception could be justified using a biopsychosocial model of chronic pain that takes into account the interactions between biological, psychological, and sociocultural factors [10]. Menstrual cycle fluctuations in ache sensitivity may help to explain sex-related differences in pain reporting in younger patients [11]. Furthermore, the biological response to pregnancy and childbearing, the physical stress of child-rearing, and perimenopausal body weight increase are additional causes of LBP [12]. Spondylarthritis, typically involving the facet joints, is common in women, and it might cause LBP [12,13]. The risk of developing spondylarthritis increases with age and/or weight [13].

Endometriosis, being a frequent condition in young women, should be considered a potential source of lumbopelvic pain. Endometriosis is commonly related to low back pain (LBP) and, therefore, often mistaken for a musculoskeletal disorder [14].

Many women are affected by the myofascial pain syndrome of the piriformis muscle, characterized by pain produced by the sciatic nerve entrapment in the piriformis muscle. Due to pelvic changes brought on by hormones and pregnancy, women are more susceptible to this pain-provoking irritation or compression of the sciatic nerve, causing radiating pain in the back of the leg [15].

Sacroiliac joint (SIJ) dysfunction problems are among the more common causes of lower back pain. The SIJ shape differs in women and men, influencing the different biomechanical joint properties. The SIJ surface area is smaller in women than in men, which can cause stress across the joint. This sex-related difference in SIJ anatomy can justify a higher risk of sacroiliac joint imbalance, especially in younger women. Pain is focused on the lower back, causing sharp pain down the thigh, and is often mistaken for sciatica [16].

The effect of body weight on LBP is controversial. While some studies have found body weight to be a weak risk factor [17], others have depicted higher body mass index (BMI) is associated with an increased risk of LBP [18].

Psychological factors, such as stress, anxiety, depression, and pain behaviors, have been reported to be associated with LBP. Conversely, the precise mechanisms underlying the associations among these factors are unclear [19].

A global review of the prevalence of LBP in the general population has shown its point prevalence is approximately 12%, with a one-month prevalence of 23%, a one-year prevalence of 38%, and a lifetime prevalence of approximately 40% [20].

Low back pain is uncommon in the first decade of life, but its prevalence increases steeply after the puberal spurt; until the age of 17 years, significant gender differences occur since it becomes more common in girls [21].

In a study conducted by Illeez et al., in which 106 children and adolescents 8–17 years were included, LBP was more common in the adolescent age group (15–16 years). There was no significant difference in gender, and non-specific LBP was the most common cause of etiology [22]. About 40% of 9–18-year-old people in high-income, medium-income, and low-income countries report suffering from low back pain [23,24].

LBP prevalence increased with age, peaked between 80 to 89 years old, and then decreased. This pattern was observed in both females and males in 1990 and 2017 [25].

Costs associated with health care and work disability attributed to low back pain vary considerably between countries and are influenced by social norms, healthcare approaches, and legislation.

The economic impact related to low back pain is comparable to other prevalent, high-cost conditions, such as cardiovascular disease, cancer, mental health, and autoimmune diseases [26].

The global trouble of low back pain will increase even further in coming decades, particularly in low-income and middle-income countries [27,28].

The differential diagnosis for low back pain is vast and should include lumbosacral radiculopathy, which describes a pain syndrome caused by the irritation or compression of nerve roots in the lower back, caused by different kinds of spinal pathologies, such as lumbar disc herniation, degeneration of the spinal vertebra, and narrowing of the foramen. Symptoms include low back pain radiating into the lower extremities in a dermatomal pattern often associated with numbness, weakness, and reflexes [29].

### 3.2. Gender-Related Differences in Musculoskeletal Pain Pathophysiology

Although the pathogenetic mechanisms underlying musculoskeletal pain are still being investigated and need more in-depth knowledge, early studies have shown that there is a gender-related difference in the pathogenesis of musculoskeletal pain based on preclinical and clinical studies. It is well known that different pain conditions that include musculoskeletal pain as a component, such as complex regional pain syndrome (CRPS), fibromyalgia, low back pain, or myofascial pain, have a higher prevalence in females [28,29,30,31,32].

Sex-related differences have been observed at multiple steps in the nociceptive signaling pathway: primary sensory afferents, spinal cord modulation, and brain processing [33].

Gene expression analysis of Dorsal Root Ganglions (DRGs) after ischemic muscle injury shows the upregulation of transient receptor potential vanilloid type 1 (TRPV1) and TRP melastatin 8 (TRPM8) [34] that appear to involve hypersensitivity to pain in females; differently, in males, it shows the upregulation of acid-sensing ion channel 3 (ASIC3) that appear linked to pain sensitization in multiple models of muscle pain, and it has been suggested that P2X5 can modulate the pH sensitivity of ASIC3 [34].

Gender differences have also been found in the mechanisms of musculoskeletal pain development in the spinal cord. Previous studies on mice demonstrated that microglia play a crucial role in the development of musculoskeletal pain in male mice but not in female mice, possibly through a mechanism dependent on the purinergic receptor, P2X4 and that the involvement of microglial pathways in pain processing was testosterone dependent. Females do not upregulate P2X4 receptors but use a microglia-independent pathway to mediate pain hypersensitivity, potentially involving T cells [33,34,35].

It is worth noting that variations in responses to muscle pain can also be attributed to gender differences in brain activation patterns, as some studies previously observed using functional magnetic resonance imaging (fMRI). These studies have demonstrated alterations in signal intensity in specific regions, the mid-cingulate cortex and dorsolateral prefrontal cortex, which exhibit a sex-dependent dimorphic pattern; this indicates the existence of significant disparities in how pain is emotionally processed between genders [36,37].

Studies to date have not yet clarified the role of gonadal hormones on the incidence of musculoskeletal pain, and further studies are needed.

### 3.3. Gender-Related Differences in LBP Conservative Management

Access to rehabilitation services is more difficult for women than men because of their family and household responsibilities [38].

Findings suggesting that women undergoing rehabilitation programs are in worse conditions than men [39] support the assumption that women claim services later, in terms of poorer health than men. Depending on family status, women and men have unequal opportunities to establish and stabilize the required behavioral and relational modifications following rehabilitation. In particular, Ref. [40] household and family tasks are mostly carried out only by women. They have significantly worse conditions for a favorable post-rehabilitation course.

Women complaining of back pain are more willing to perform voluntary physical therapy activities than men [41].

It is known from cardiac rehabilitation that it is more important for women to decide for themselves what physical exercises they want to do; moreover, women are significantly less willing to perform exhaustive or pain-producing activities [42].

This aspect also plays a key role in orthopedic rehabilitation. It is supported by a study from Finland, which concludes that women seem to benefit less from activating rehabilitation measures geared to sport and exercise than from traditional procedures characterized mainly by passive physical therapy measures (massage, electrotherapy, etc.) and light or passive exercises [43].

The clinical outcome of patients undergoing rehabilitation is significantly conditioned by several factors that predict treatment response relative to a baseline condition. Baseline pain intensity and baseline pain-related disability predicted the modification in pain intensity for both women and men [44]. Baseline pain-related disability and duration of symptoms, on the other hand, predicted the modification of pain intensity for women only [44]. Finally, stabilization exercise and leg pain predicted change in pain-related disability for male patients [44].

### 3.4. Gender-Related Differences after Major Vertebral Surgery

LBP prevalence increases during aging. Therefore, the progressive increase in age might justify the increasing rate of surgeries to treat back pain. Considering the significant increase in the prevalence of back pain over time, it is understandable that there are similar trends in increasing rates of surgeries to treat it. However, sometimes surgery is unable to give long-term pain relief, offering only temporary relief of the patient’s pain [45]. It is reported that a relevant number of back surgeries, comprised between 20 and 40%, could be perceived as unsuccessful by patients [45].

Failed back surgery syndrome is the medical term used to indicate this lack of success. The International Association for the Study of Pain defines failed back surgery syndrome (FBSS) as a lumbar spinal pain of unknown origin that persists after surgery or appears in the postoperative period [46].

Several studies have sought to identify factors that might predict the success of surgery [47]. These generally include work-related, lifestyle-related, anatomical, morphological, and/or genotypic factors related to sex/gender and different ethnicities that can impact the treatment of different pathologies [48].

Nonetheless, there are times when back surgery is a viable or necessary option to treat serious musculoskeletal injuries or nerve compression.

The National Institute of Neurological Disorders and Stroke (NINDS) lists some of the surgical options for low back pain. But NINDS also highlights that “there is little evidence to show which procedures work best for their particular indications” [49].

Few studies in the literature have examined gender differences in patient-rated outcomes about painful disorders of the spine and the outcome of their surgical treatment.

Yang et al. [50] showed that patients undergoing percutaneous kyphoplasty (PKP) can immediately obtain pain relief and activity function improvement. PKP is an effective and safe minimally invasive surgery currently used for both female and male patients [50]. The pain improvement was recorded using the visual analog scale (VAS). Immediately after the operation and one-year follow-up, the mean VAS was significantly lower than that before surgery, and there was no difference between the groups at each time [50,51,52].

Gehrchen et al., in a retrospective study, reported that the female gender is an independent risk factor for nonoptimal outcomes after lumbar fusion [53]. A randomized controlled trial observed by Erkman et al. that the female gender was also associated with worse postoperative results [54].

However, a prospective clinical study conducted by the Swedish National Spine Register on 4780 patients suffering from lumbar degenerative disc disease and chronic low back pain depicted that female patients had worse pain and function preoperatively but then improved significantly more than males after surgery [55].

Salamanna et al. analyzed in a review the studies considering sex- and gender-related differences in patients treated for lumbar degenerative diseases (disc degeneration, disc herniation, and spondylolisthesis) [56]. More than half of the studies reported a worse postoperative outcome in terms of pain, disability, health-related quality of life (HRQoL), and complications in women, while the remaining reported worse clinical outcomes in terms of satisfaction and HRQoL in male patients [56]. In studies examining pain, disability, and HRQoL differences between females and males, the complexity of evaluating these parameters, which include many clinical signs and subjective outcomes, must be considered [56].

The prolonged opioid consumption in the postoperative period is probably the reason why this gender is associated with the worst postoperative state [57]. This systematic review reported that women were also at increased risk of complications (urinary tract infection, readmission for pain or neurological symptoms, adjacent segment disease) and consequent longer length of stay (LOS) following spinal fusion surgery for lumbar degenerative disease [57,58,59,60,61,62,63,64,65].

MacLean et al. have observed that in the postoperative period, female patients showed worse absolute pain, disability, and quality of life but showed equal or greater interval change compared with men in lumbar degenerative disease [60].

## 4. Limitations

The papers included in the present review have been analyzed for different aspects of LBP (epidemiology, pathophysiology, conservative treatment, and surgery). Hence, the results do not show the whole population, but only specific subgroups that will benefit from a tailored diagnostic and therapeutic approach.

In some papers, the terms “sex” and “gender” are used as synonyms, but it is important to differentiate the clinical meaning of these two words to correctly apply gender medicine in clinical practice.

## 5. Conclusions

Sex- and gender-related factors should be carefully assessed and addressed in patients suffering from LBP to improve clinical outcomes and patients’ quality of life.

Clinical researchers should pay more attention to gender-specific indicators to develop gender-based risk-stratification models.

The findings of this review might be beneficial in daily clinical practice to improve the management of patients suffering from LBP by providing personalized treatment, able to significantly reduce the impact of this type of pain on the patient’s quality of life.

## Data Availability

Study data are available upon request to the corresponding author.
